# The Multifaceted Role of the Inflammasome in Inflammatory Bowel Diseases

**DOI:** 10.1100/tsw.2011.139

**Published:** 2011-07-28

**Authors:** Donata Lissner, Britta Siegmund

**Affiliations:** Medizinische Klinik I, Charité – Universitätsmedizin Berlin, Campus Benjamin Franklin, Berlin, Germany

**Keywords:** cytokines, inflammatory bowel diseases, inflammasome

## Abstract

Inflammasomes are intracellular multiprotein complexes that coordinate the maturation of interleukin (IL)-1β and IL-18 in response to pathogens and metabolic danger. Both cytokines have been linked to intestinal inflammation. However, recently evolving concepts ascribe a major role to the inflammasome in maintaining intestinal homeostasis. This review recapitulates its position in the development of inflammatory bowel disease, thereby outlining a model in which hypo- as well as hyperfunctionality can lead to an imbalance of the system, depending on the specific cell population affected. In the epithelium, the inflammasome is essential for regulation of permeability and epithelial regeneration through sensing of commensal microbes, while excessive inflammasome activation within the lamina propria contributes to severe intestinal inflammation.

Interleukin (IL)-1β and IL-18, both belonging to the IL-1 family, represent two crucial cytokines involved in the pathogenesis of both acute and chronic inflammatory disorders[1]. Because of their potency to cause local and systemic inflammation, close control mechanisms for their secretion are required. Therefore, maturation of these cytokines is a process of multiple consecutive steps, each of them displaying potential regulatory units[2]. Both are, after a specific first signal, produced as inactive proforms and undergo subsequent maturation through cleavage into their active forms after the essential second signal. For the control of this second hit, a protein complex called the inflammasome is mostly accountable, since it mediates the ultimate step, i.e., the activation of caspase-1, which then cleaves pro–IL-1β and pro–IL-18 into their mature forms[3].

The first description of what is now referred to as IL-1 dates back a long time[4]. In fact, in the early 1940s, a leukocyte-derived pyrogenic protein was described, which would be purified[5] and finally cloned[6] many years later. The activating enzyme caspase-1, at that time called IL-1β-converting enzyme (ICE), was identified and cloned in 1992[7]. In comparison, the inflammasome and thus the molecular mechanisms responsible for the activation of caspase-1 have long remained poorly understood and have just recently been elucidated[8].

This review recapitulates recently evolving concepts of inflammasome activation and its relevance *in vivo*, concentrating on its complex and controversially discussed role within intestinal inflammation and mucosal injury. Based on the literature, a model will be outlined in which the inflammasome, as an important member of the innate immune system, plays a decisive role in maintaining intestinal homeostasis, where hypo- as well as hyperfunctionality can result in an imbalance of the system, depending on the specific cell population affected.

## THE INFLAMMASOME

Inflammasomes are high-molecular-weight platforms in the cytosol of diverse cell types. Because they represent a component of the innate immune system, most studies identifying inflammasome-expressing cells have focused on derivatives of the myeloid lineage, such as macrophages and dendritic cells[9,10]. However, other cell types have been identified that contain fully functional inflammasomes as well, such as keratinocytes[11], epithelial cells[12,13,14], and adipocytes[15]. Inflammasomes are compounded by several self-oligomerizing proteins whose exact compositions differ depending on the distinct inflammasome subtype. The best characterized inflammasome subtype in intestinal inflammation is the NLRP3 inflammasome, which consists of the NLRP3 (NACHT, LRR, and PYD domains containing protein 3) protein, the ASC (apoptotic speck-like protein containing a CARD) adaptor, and pro–caspase-1 (see Fig. 1).

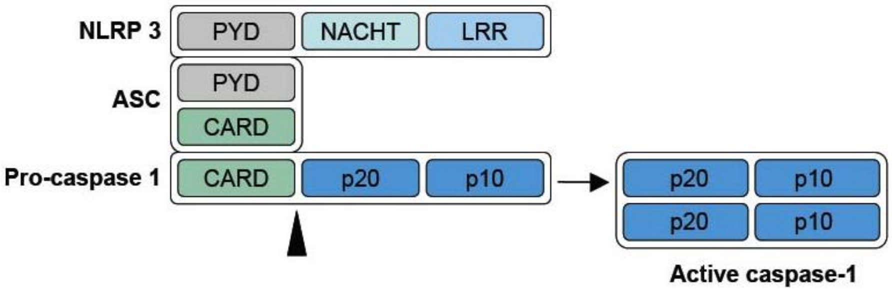

**FIGURE 1.** The NLRP3 inflammasome. The NLRP3 inflammasome consists of the NLRP3 protein, the ASC adaptor, and pro–caspase-1. Upon stimulation, NLRP3 and ASC adhere via their PYD domains, whereas ASC and caspase-1 adhere via their CARD domains. After autocleavage of the inactive CARD domain from the subunits (p20 and p10), caspase-1 binds to a second monomer, resulting in the active p10/p20 tetramer.

As explained above, two signals are required for the secretion of active IL-1β and IL-18 (see Fig. 2). The first signal, which can, among others, occur through Toll-like receptors (TLR) in response to pathogen-associated molecular patterns (PAMPs) or IL-1β itself, induces the transcription and subsequently translation of the inactive proforms via nuclear factor “kappa-light-chain-enhancer” of activated B cells (NF-κB) activation. The second signal results in inflammasome activation and is required for the cleavage of the proforms into active cytokines. When NLRP3, also known as cryopyrin, gets activated, its oligomerization leads to PYD domain clustering, thus recruiting ASC. Through its CARD domain, ASC binds pro–caspase-1, which ultimately mediates the activating cleavage process[16]. Caspases are cysteine proteases, which are produced as inactive zymogens and need to undergo proteolytic activation. Different subclasses of caspases have been described, with some of them being involved in the induction of apoptosis and others, like caspase-1, displaying proinflammatory properties[17]. The inactive proform pro–caspase-1 is composed of the CARD domain and two subunits (p20 and p10). After heterodimerization, the CARD domain and the subunits are released by autocleavage, resulting in the active p10/p20 tetramer[7] (see Fig. 1).

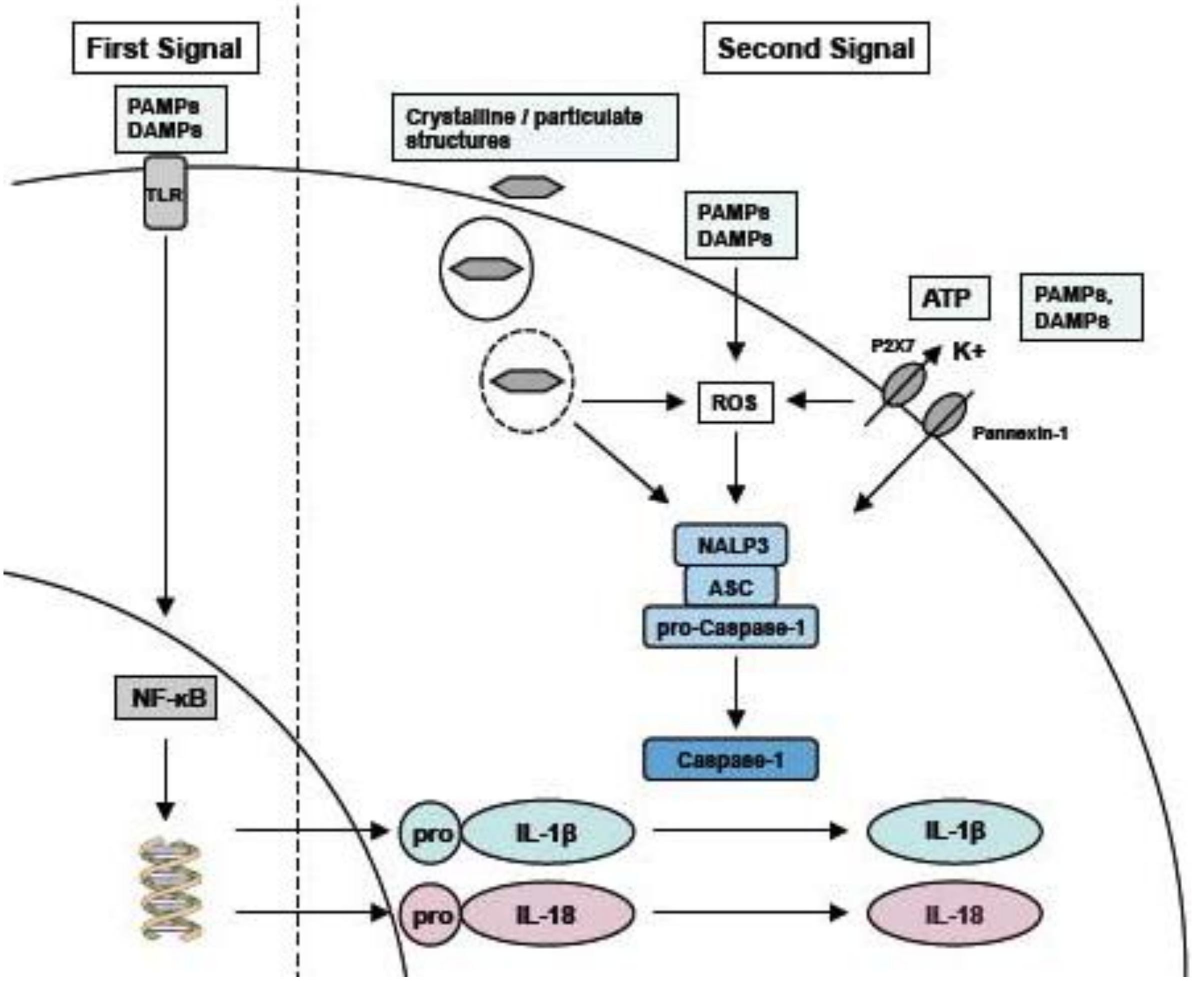

**FIGURE 2.** First and second signal for inflammasome activation. For inflammasome activation, two signals are required. The first one, which can be conducted through PAMPs or DAMPs, leads to the NF-κB–dependent transcription and translation of pro–IL-1β and pro–IL-18. For the second signal, several mechanisms have been suggested. Extracellular ATP leads to a P2X7-dependent pore formation in the cell membrane, which entails a potassium efflux from the cell. This efflux leads to emplacement of large-pored pannexin-1 channels into the membrane, through which PAMPs and DAMPs can now access the cell and directly activate NLRP3. Larger molecules, such as crystalline or particulate ligands, are incorporated by phagocytosis. After rupture of the phagolysosome, they can again directly bind and activate the inflammasome. ROS are generated after exposure to PAMPs and DAMPs, but also through the ATP-dependent potassium efflux and lysosomal rupture, and can activate the inflammasome via the ROS-sensitive ligand, thioredoxin-interacting protein. Most likely, all mechanisms occur concomitantly. The second signal results in the cleavage of pro–IL-1β and pro–IL-18 into the active forms. ATP, adenosine triphosphate; DAMPs, danger-associated molecular patterns, ROS, reactive oxygen species.

For NLRP3 activation, several mechanisms have been proposed (see Fig. 2). One suggests that extracellular adenosine triphosphate (ATP) induces a P2X7-dependent pore formation in the cell membrane, thus mediating a shift from intra- to extracellular potassium[18]. This, in turn, triggers the formation of pannexin-1 channels into the membrane, allowing the higher molecular PAMPs and danger-associated molecular patterns (DAMPs) penetration into the cell and thus direct activation of NLRP3[19]. The other suggested mechanism describes phagocytosis of crystalline or particulate ligands, which access the cell after the rupture of the phagolysosomes, where they can again directly bind and activate the inflammasome[20]. A third model provides data indicating that reactive oxygen species (ROS), generated after exposure to PAMPs and DAMPs, can activate the inflammasome via a ROS-sensitive ligand, thioredoxin-interacting protein[21,22]. This is strongly underlined by the finding that chemically induced ROS blockade potentially inhibits inflammasome activation.

Since one fundamental function of the inflammasome is the coordinated response to pathogen infections, its dominant activators are whole microorganisms and PAMPs. Potential identifiable pathogens include fungi (*Candida albicans*, *Saccharomyces cerevisiae*)[23], bacteria (*Staphylococcus aureus*, *Listeria monozygotes*)[24], and viruses (influenza virus, adenovirus)[25,26]. Apart from pathogens, exogenous mechanical factors, such as ultraviolet B irradiation[11], and particulate ligands, such as silica[27] and asbestos[28], can equally lead to inflammasome activation and IL-1β release. In recent years, the spectrum of activating factors has been broadened even more, since several host-derived molecules (ATP, hyaluronan) and metabolic stress, such as hyperglycemia[29] and free fatty acids[30], could been identified to potentially induce inflammasome activation. Consequently, the inflammasome, although a representative of the innate immune system, serves not only as a sensor for infections, but also for metabolic danger.

## INFLAMMASOME-ASSOCIATED HUMAN DISEASES

There is evidence for a number of inflammatory diseases to be associated with inflammasome dysfunction. For some of them, hyperfunctional genetic mutations within the NLRP3 gene are proven and lead to their name, cryopyrin-associated periodic syndromes (CAPS)[31]. Counted among the so-called autoinflammatory diseases, CAPS comprise Muckle-Wells syndrome (MWS), familial cold autoinflammatory syndrome (FACS), and neonatal-onset multisystem inflammatory disease (NOMID). For each of them, a rapid and nearly complete response to the treatment with anakinra, a soluble selective IL-1 receptor antagonist, is characteristic[32,33]. Other autoinflammatory diseases display similar clinical manifestations, but these patients do not carry the described genetic mutation, such as familial Mediterranean fever, hyper IgD-syndrome, adult and juvenile Still's disease, or Behçet's disease. These diseases are equally characterized by an immediate responsiveness to IL-1 blockade[34,35,36].

In addition to these rare autoinflammatory diseases, there is increasing evidence for the involvement of the inflammasome pathway on other, more common, medical conditions for which hereditary as well as acquired components seem likely. One such disease is rheumatoid arthritis (RA), for which the combination of *NLRP3* and *CARD8* polymorphisms represents a risk factor[37]. IL-1 receptor blockade is an approved strategy to decrease disease severity and the destructive joint process in RA, and is comparable to other treatment options, such as tumor necrosis factor (TNF)-α blockade[38,39]. In addition, there is emerging evidence for the crucial role of IL-1 on diseases associated with the metabolic syndrome, which have long not been seen as inflammatory diseases. Namely, type 2 diabetes mellitus has been shown to develop in an inflammasome-dependent manner[15,29,40]. The functional impact is strongly underlined by the clinical study where the blockade of IL-1 with anakinra improved hyperglycemia and β-cell secretory function, and reduced markers of systemic inflammation[41]. Furthermore, IL-1 blockade has proven highly effective in gout[42,43], another inflammatory condition associated with the metabolic syndrome. In this case, free fatty acids have proven to be potent inducers of gout attacks through inflammasome activation[30].

## GENETIC MUTATIONS WITHIN THE INFLAMMASOME PATHWAY ASSOCIATED WITH INFLAMMATORY BOWEL DISEASES

In genome-wide association studies, single nucleotide polymorphisms (SNPs) in the *NLRP3* gene have recently been linked to the susceptibility to develop Crohn's disease (CD)[44], which was confirmed by another group reporting a higher risk for men carrying polymorphisms within the *NLRP3* and *CARD8*

genes[45]. Remarkably, these findings were not confirmed by a large study from the U.K. comprising approximately 13,000 CD patients[46], which may be explained by regional differences in genetic background. Former studies had reported mutations within *IL18* itself[47] or the corresponding receptor[48] to carry a higher risk for developing inflammatory bowel diseases (IBD). Taken together, these studies provide evidence for a hypofunctional inflammasome to increase the risk for developing IBD, a finding that reminds us of another susceptibility gene for CD, the *NOD2/CARD15* loss-of-function mutation[49]. Belonging to the same NLR family, NOD2 and NLRP3 are close relatives that share the feature of being intracellular pattern recognition receptors of the innate immune system to sense microbes and pathogens invading the cell. In both cases, inadequate sensing of pathogens is associated with chronic intestinal inflammation. Considering these findings, the exact comprehension of the inflammasome pathway within the complex interaction between microbiota, barriers, and the intestinal immune system is the key to understanding its role in the development of chronic mucosal inflammation and, thus, IBD.

## INFLAMMASOME AND INFLAMMATORY BOWEL DISEASES

IBD represent a chronic inflammatory condition of the gut, for which the innate as well as the adaptive immune system play a crucial role. Although the exact mechanisms are not fully understood, the concept of an inadequate immune response against commensal microbiota in the gut lumen is widely accepted[50]. Long before the inflammasome itself was characterized, the downstream cytokines IL-1β and IL-18 were considered to be causally related to IBD development. The first evidence for the involvement of IL-1β was described in 1989, when Mahida et al. reported enhanced production of this cytokine in mononuclear cells isolated from the actively inflamed mucosa of IBD patients[51]. Similar results were later published for IL-18, reporting increased expression of IL-18 on mRNA and protein level in the affected mucosa of IBD patients[52]. The up-regulated cytokines where localized in classical immune cells (macrophages and dendritic cells), but also in intestinal epithelial cells (IEC). These findings where supported by others who confirmed the up-regulation of IL-18 in the mucosa of CD patients, also describing a correlation with the degree of inflammation[53]. The concept of IL-1β and IL-18 being the "bad guys" in mucosal inflammation was underlined by *in vitro* studies where the addition of IL-1β to epithelial cells altered tight junctions and thus intestinal permeability[54]. However, the functional relevance of such concepts must always be thoroughly evaluated in *in vivo* studies, where in this case, no consistent affirmation could be achieved. Quite the contrary turned out to be true in animal studies, depending on the specific approach applied – cytokine blockade or gene knockout strategies.

### *In vivo* Studies: Neutralization Strategies

The first evidence for effective IL-1β inhibition was reported *in vivo* by applying a soluble IL-1 receptor antagonist (IL-1Ra) to suppress immune-complex colitis in rabbits[55]. In this model, the ratio of endogenous IL-1Ra seemed to be crucial: Administration of a specific IL-1Ra antibody increased colonic inflammation and mortality[56]. Evidence for protection from colitis through anti–IL-18 strategies was provided later by studies where, through the administration of anti–IL-18 antibodies, a dramatic attenuation of mucosal inflammation in trinitrobenzene sulfonic acid (TNBS)–induced[57] or dextran sulfate sodium (DSS)–induced[58,59] colitis could be achieved. Other approaches, either by blocking the conversion of pro–IL-1β or pro–IL-18 into the active cytokines using a specific caspase inhibitor[60,61,62], or by applying IL-18 antisense mRNA through an adenovirus[63], indicated equal effectiveness in the amelioration of experimental colitis. In these studies, the cytokine production was localized to IEC and lamina propria immune cells, thus reinforcing the findings of human colonic specimens.

## *In vivo* Studies: Knockout Mice

However, when looking at *in vivo* studies accomplished in knockout animals for genes involved in inflammasome signaling or downstream pathways, this concept could not be proven. Contrary to what was assumed after the promising IL-18 blockade, *Il18*$^{-/-}$ and *Il18r1*$^{-/-}$ mice developed more severe DSS-induced colitis, with higher mortality rates, compared to their wild-type counterparts[64]. Similar effects have subsequently been proven for IL-1 receptor signaling in a model of infectious colitis[65]. However, this study could not reveal differences when assessing chemically induced colitis. Several recent studies provide explicit data that *Nlrp3*$^{-/-}$ mice are more prone to develop colitis[14,66,67]. The same is true for *Asc*- and *Caspase1*-deficient mice[68]. Only one study reports the opposite effect, as they describe an Nlrp3-dependent mechanism for the development of DSS colitis[69]. Yet in all other studies, the genetically altered animals exhibited dramatic colitis after DSS or TNBS treatment, with high mortality and morbidity rates. They display increased intestinal permeability with enhanced bacterial translocation[14]. Since transplantation of wild-type bone marrow into *Nlrp3*$^{-/-}$ mice confirmed these results, Nlrp3-expression in nonhematopoetic cells, such as IEC, seems to be crucial. Interestingly, *Nlrp3*$^{-/-}$ mice without DSS treatment do not exhibit any signs of colitis, implying that inflammasome deficiency alone does not result in the imbalance of the intestinal homeostasis. This pathway rather seems to be involved in the reconstitution of homeostasis after an injury has occurred. In line with this consideration, *Caspase1*$^{-/-}$ mice display impaired IEC proliferation after DSS-induced mucosal injury, as seen by proliferation assays[68]. A causal correlation of the absent epithelial regeneration to impaired IL-18 maturation has been proven, since high serum and tissue concentrations of this cytokine have been found in DSS-treated wild-type mice, but not in knockout mice, whereas no difference was seen regarding IL-1β levels. This was further emphasized by the reversibility through exogenous IL-18 administration[14,68]. Furthermore, *Nlrp3*$^{-/-}$ mice featured reduced amounts of colonic defensins[67], which naturally confer antimicrobial activity. This is of clinical importance, since a deficiency in mucosal α-defensins type 4 and 5 has been linked to the pathogenesis of small-bowel CD[70].

The exact mechanism by which IL-18 is able to prevent colitis still remains a matter of debate. Beyond dispute is the ability of IL-18 to induce interferon γ (IFNγ) production in T cells[71], which in turn regulates IEC proliferation and reconstitution of the epithelial barrier, thus protecting against colitis[72]. Whether there are other cytokines concurrently involved in this pathway or whether IL-18 exerts its regenerative effects through initiating other cascades at the same time needs to be clarified.

Remarkably, other more ubiquitously occurring signaling pathways of the innate immune system have also been identified as pivotal for the maintenance of healthy mucosa. IL-1β, IL-18, and all TLR signaling, except for TLR3, is mediated by a cytoplasmic adaptor molecule, the myeloid differentiation protein (Myd88)[73]. Mice deficient for this fundamental signaling molecule are highly susceptible to chemically induced colitis[74]. The same accounts for mice deficient in TLR2[75] and TLR4[76]. Furthermore, stimulation of the Nod2 receptor by muramyl dipeptide (MDP) protects mice from developing severe colitis[77], and *Nod2*$^{-/-}$ mice display an altered colonic microbiota when compared to wild-type mice[78], an observation equally seen in *Nlrp3*$^{-/-}$ mice[67]. Patients carrying NOD variants display more bacterial translocation and an increased presence of endotoxin within the lamina propria compared to controls[79]. Together, these results imply that sensing of commensal microbiota, adequate pathogen clearance, and an immune response to bacterial translocation by the innate immune system are essential for inducing and sustaining intestinal homeostasis.

Thus the results from pharmacological blocking strategies are not in complete agreement with newer studies investigating gene knockout approaches. Possible explanations are multifaceted. One is that neutralizing therapies, either with antibodies, antagonists, or enzyme blockades, are never able to fully inhibit the desired target in all compartments and cells of the body. The pharmacological features constrict availability, distribution, and half-life of each substance. Also, every therapy carries side effects, thus biasing the study outcome. In genetic knockout approaches, however, every cell in the body carries the same mutation, thus ensuring that no protein synthesis of the designated target occurs at all. Therefore, these studies are often considered as being more appropriate to study functional gene aspects.

However, as mentioned above, genetic studies can also disagree regarding their results. One has to keep in mind that various factors, such as the genetic background of the test animals, their breeding and environment, and the specific approach to induce colitis, significantly affect the disease course, therefore explaining such discrepancies.

Another aspect is that the effect of the inflammasome pathway on intestinal inflammation strongly depends on the specific cell population affected (see Fig. 3). As explained above, its activation in epithelial cells, most notably through maturation of IL-18, is required for induction and maintenance of intestinal homeostasis, since pivotal steps such as sensing of the commensal microbiota, bacterial clearance, and defensin synthesis, are conducted via this tightly controlled signaling pathway. However, when the epithelial barrier is disrupted, as occurs in IBD, microbes and antigens access the lamina propria, where they are detected by macrophages and dendritic cells through pattern recognition receptors (PRRs). Inflammasome activation below the epithelium, such as in macrophages, dendritic cells, and even adipocytes in the mesenteric fat tissue, of which inflammasome expression has been shown for all, may have deleterious effects on mucosal inflammation. In this case, IL-1β is probably the critical cytokine, explaining why neutralizing therapies exert beneficial effects in this scenario. These cells can be activated via “classical” stimulators, such as pathogens or their associated patterns, which access the actual sterile area through bacterial translocation and epithelial leakage. However, other inflammasome-triggering factors must equally be taken into account. As discussed above, metabolic stress is emerging as a crucial inflammasome activator and has recently been linked to a number of diseases associated with the westernized lifestyle[40]. It can be assumed that this axis might also be connected to the development of IBD, considering the rising incidence rates in developed countries[80] and the aggravated course in obese IBD patients[81]. However, this hypothesis requires further evaluation regarding its clinical significance.

## CONCLUSION

It has long been stated that increased secretion of the inflammasome effector cytokines IL-1β and IL-18 is associated with intestinal inflammation and the increased risk of developing IBD. However, the regulatory role of NLRP3 in intestinal inflammation is rather multifaceted. Our traditional understanding of the innate immune system implies that it serves as a first response to microorganisms and injuries, resulting in appropriate defense mechanisms and inflammation. Yet, our understanding has to be broadened, since the NLR proteins NOD1 and 2 and NLRP3 are essential mediators of regenerative processes and proliferation within the epithelium, most likely through the maturation of IL-18. In this compartment, their absence rather than their overproduction is deleterious. We therefore propose a model in which inflammasome hypo- as well as hyperfunctionality can lead to an imbalance of the intestinal homeostasis, depending on the specific cell population affected: In the epithelium, the inflammasome is essential for regulation of permeability and epithelial regeneration through sensing of commensal microbes; however, excessive inflammasome activation within the lamina propria contributes to severe intestinal inflammation.

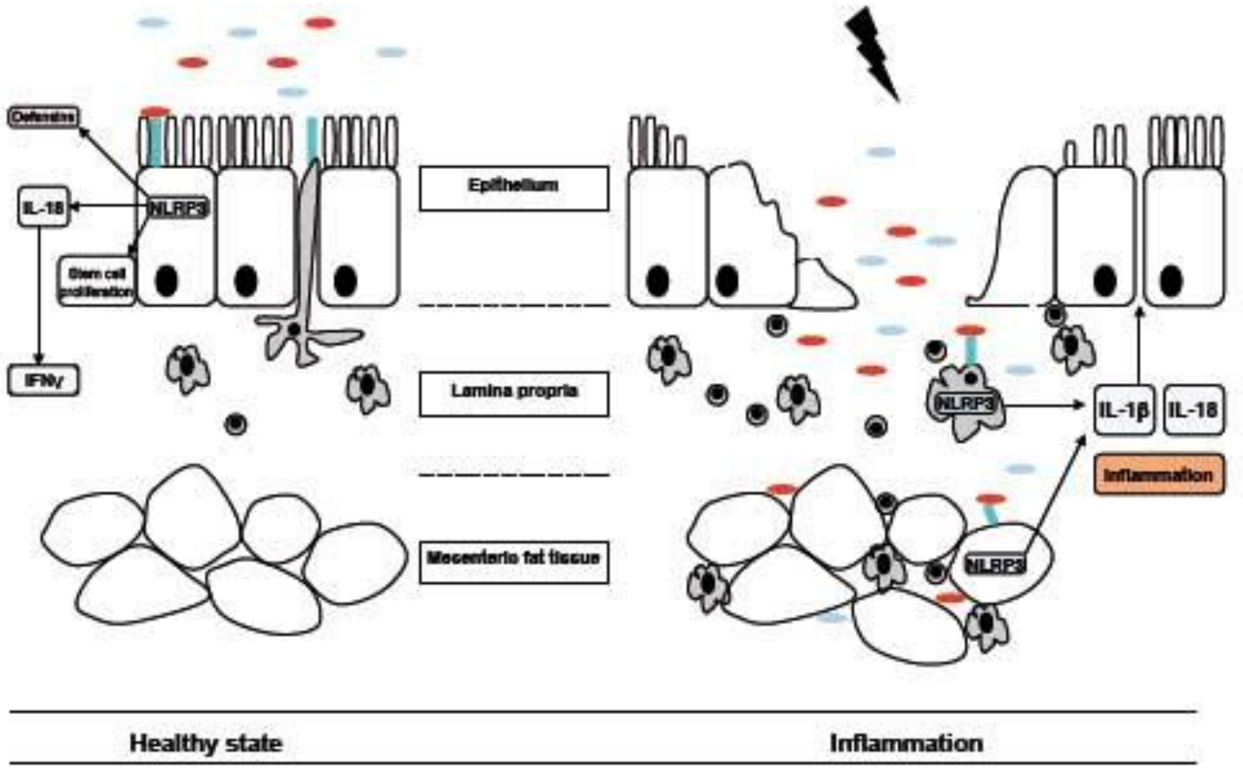

**FIGURE 3.** Model of inflammasome activation depending on the affected cell type. In the healthy state, the epithelium senses commensal microorganisms through the NLRP3 inflammasome. Thus, mainly IL-18 causes stem cells from the base of the crypts to mature to IECs, thus contributing to maintenance of intestinal homeostasis. IL-18 also induces the synthesis of defensins as well as of IFNγ, both regulating the reconstitution of the epithelial barrier. However, when the epithelial barrier is disrupted, microbes and antigens gain access to the lamina propria and the mesenteric fat tissue, which results in a massive invasion of immune cells into this area. Through PRRs, inflammasome activation occurs in macrophages, dendritic cells, and adipocytes. Here, the maturation of IL-1β probably multiplies the local inflammatory response and leads to further increase in intestinal permeability through alteration of tight junctions.

## ACKNOWLEDGMENT

This study was supported by the Sonderforschungsbereich 633 (to DL and BS) and DFG SI 749/6-1 (to BS).
